# The efficacy and further functional advantages of random-base molecular barcodes for absolute and digital quantification of nucleic acid molecules

**DOI:** 10.1038/s41598-017-13529-3

**Published:** 2017-10-19

**Authors:** Taisaku Ogawa, Kirill Kryukov, Tadashi Imanishi, Katsuyuki Shiroguchi

**Affiliations:** 1 Laboratory for Integrative Omics, RIKEN Quantitative Biology Center (QBiC), 6-2-3 Furuedai Suita, Osaka, 565-0874 Japan; 2 Biomedical Informatics Laboratory, Department of Molecular Life Science, Tokai University School of Medicine, 143 Shimokasuya, Isehara, Kanagawa 259-1193 Japan; 3 Laboratory for Immunogenetics, RIKEN Center for Integrative Medical Sciences (IMS), 1-7-22 Suehiro-cho, Tsurumi-ku, Yokohama 230-0045 Japan; 4 JST PRESTO, 4-1-8 Honcho, Kawaguchi, Saitama 332-0012 Japan

## Abstract

Accurate quantification of biomolecules in system-wide measurements is in high demand, especially for systems with limited sample amounts such as single cells. Because of this, digital quantification of nucleic acid molecules using molecular barcodes has been developed, making, e.g., transcriptome analysis highly reproducible and quantitative. This counting scheme was shown to work using sequence-restricted barcodes, and non-sequence-restricted (random-base) barcodes that may provide a much higher dynamic range at significantly lower cost have been widely used. However, the efficacy of random-base barcodes is significantly affected by base changes due to amplification and/or sequencing errors and has not been investigated experimentally or quantitatively. Here, we show experimentally that random-base barcodes enable absolute and digital quantification of DNA molecules with high dynamic range (from one to more than 10^4^, potentially up to 10^15^ molecules) conditional on our barcode design and variety, a certain range of sequencing depths, and computational analyses. Moreover, we quantitatively show further functional advantages of the molecular barcodes: the molecular barcodes enable one to find contaminants and misidentifications of target sequences. Our scheme here may be generally used to confirm that the digital quantification works in each platform.

## Introduction

In the modern big data era of biology, accurate quantification of biomolecules in system-wide measurements is required as the quality of the analysis depends highly on the initial raw data. Because of this, digital quantification of nucleic acid molecules using DNA tags (so-called “primer IDs”^1^, “unique molecular identifiers”^2^, or “molecular barcodes”^3^) has been previously developed. This technique has been used for many applications in next generation sequencing platforms, such as gene expression analysis by RNA sequencing (RNA-Seq)^2–7^, iCLIP (individual-nucleotide resolution UV cross-linking and immunoprecipitation)^8^, antibody repertoire analysis^9^, bacterial 16 S rRNA gene analysis^10,11^, and ChIP-nexus (chromatin immunoprecipitation experiments with nucleotide resolution through exonuclease, unique barcode and single ligation)^12^. These methods enable one to determine the absolute number of molecules in a given sample accurately in a digital manner even in the presence of noise and/or bias in the measurement system. RNA-Seq using molecular barcodes, i.e., digital RNA-Seq (dRNA-Seq)^3^ or quantitative RNA-Seq.^13^, is one of the most widely used applications of digital counting. Since dRNA-Seq works well even for small sample sizes, it has often been used for single cell gene expression analyses. In such measurements, the detection limit is important because single cells have been shown to have many low-copy RNAs^13,14^, and the detection limit indicates that there are many potentially undetected low-copy RNAs, which may affect the subsequent interpretation of biological phenomena. Therefore, an investigation into the efficacy of barcodes for absolute and digital quantification is crucial, as the barcode system used determines the detection limit of nucleic acid quantification. Moreover, the simultaneous efficacy of a barcode’s ability to count high copy number species is also important because, for example, random-base barcodes may be used to label a few thousand virus RNAs^1^, and to identify thousands of cells in a study of high-throughput single cell RNA-Seq where the barcodes were used to distinguish individual cells in a single sequencing run^7^.

**Figure 1:**
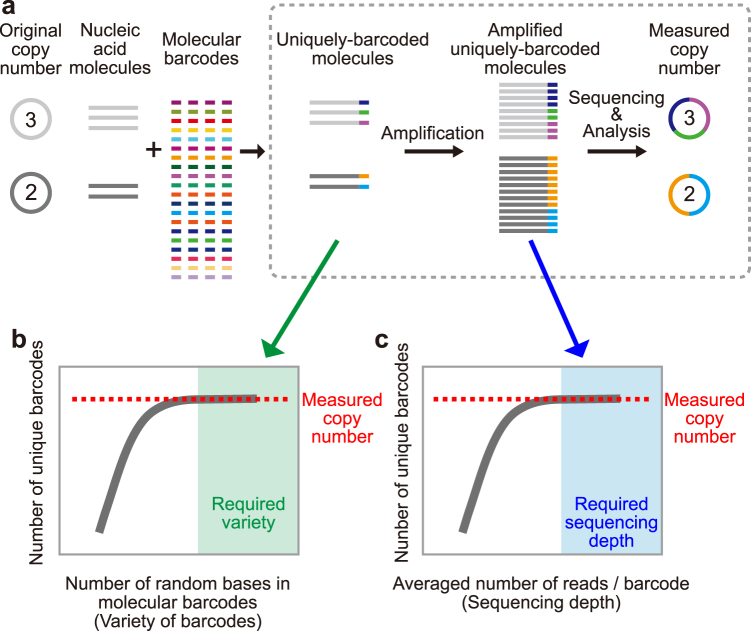
Digital quantification of nucleic acid molecules and its efficacy. (**a**) Scheme of digital counting. A molecular barcode is attached uniquely to each target molecule. After amplification, both the target section and the barcode section are sequenced. The copy number is determined by the number of unique barcodes instead of the number of reads. Dotted frame; experimental design in this study. (**b**) The first requirement for accurate digital counting: each target molecule should be labeled by different barcodes. If the measured number of unique barcodes is constant as the variety of barcode sequences increases, that range of the variety of barcode sequences fulfills the first requirement. (**c**) The second requirement for accurate digital counting: all barcodes attached to the target molecules should be detected (at least one read). If the measured number of unique barcodes is constant as the sequencing depth increases, that range of sequencing depths fulfill the second requirement.

The general procedure of digital quantification of nucleic acid molecules is as follows (Fig. 1a): (i) Each RNA or (complementary) DNA ((c)DNA) is uniquely tagged by an externally-added DNA (molecular barcode)^1–3^ that contains a large variety of sequences. (ii) The barcoded (c)DNA (generated from RNA when starting from RNA) is amplified. (iii) Both target and barcode sequences of the amplified barcoded (c)DNAs are sequenced in tandem. (iv) The number of unique barcodes, rather than the number of amplified molecules (so called “reads”), is quantified for each target (or gene) in order to provide the absolute copy number of the original target (i.e., pre-amplified RNA or (c)DNA) before amplification as proposed theoretically^15^. This scheme is able to remove the effect of noise and/or bias generated in various steps during measurement of the system, such as from amplification, sequencing, and/or analysis. To ensure that the digital counting system works adequately, one must use a large variety of barcode sequences such that each target molecule is guaranteed (or nearly guaranteed) to be uniquely tagged so that the measured number of unique molecular barcodes is equal to the number of given target molecules^16,17^ (the first requirement below). Furthermore, it is empirically thought that sufficient sequencing depth is necessary for accurate counting (the second requirement below)^18,19^.

In the digital counting scheme, typically two types of barcode designs have been used: sequence-restricted barcodes (each barcode sequence is individually designed) and non-sequence-restricted barcodes (occasionally called a “random”-base barcode). When sequence-restricted barcodes were used previously, the required varieties of barcode sequences for accurate quantification was estimated by theoretical calculation^16^, and the capability of this technique for the absolute quantification of barcoded molecules was confirmed experimentally^3,16^. However, there is a disadvantage in using sequence-restricted barcodes: many different individually-designed barcode sequences must be prepared for high dynamic range measurements, which is not cost effective. In order to minimize cost while also increasing the dynamic range of counting, random (or pseudo-random)-base barcodes have been used instead^2,4–9,11,12,18,20^. In this case as well, it should be determined that the sequence variety in the barcode set is sufficient^17,18^. However, unlike for sequence-restricted barcodes, this investigation is not trivial simply because of sequence changes in the barcode due to sequencing and/or amplification errors; a newly generated barcode sequence from one of these errors may become a false positive^21^. Namely, the errors cause overestimation of the number of molecules in a sample (note that in the case of sequence-restricted barcodes, all used barcode sequences are known meaning that all unused barcode sequences are also known so that the sequences resulting from errors can be identified and removed). This problem is approached by removing errors using computational analyses based on the reasonable assumption that similar barcode sequences generated through errors originated from the same original barcode sequence. Moreover, Sudbery and collaborators recently showed the efficacy of a random-base UMI (molecular barcode) based on computational analyses by modeling errors for a limited dynamic range (up to 100 molecules)^22^. However, the efficacy of random-base molecular barcode for accurate digital counting has not been clearly shown, particularly in a quantitative sense^7,20^ and at high dynamic range, based on experiments that obviously may include effects not present in theoretical models.

Here, we show experimentally that random-base molecular barcodes can be utilized for digital quantification of the absolute number of barcoded DNA molecules when using our specific barcode design and after computational analyses. In order to investigate the efficacy of the barcode itself by removing other effects such as barcode attachment and/or reverse transcription which may vary in different applications, we used synthesized DNA molecules that contain barcode sequences, and quantified them by sequencing for amplified molecules (dotted frame in Fig. 1a). For accurate digital counting, we quantitatively investigated the two requirements above; (i) that a large enough set of barcode sequences compared with the number of given molecules was used (described above) (Fig. 1b), and (ii) that sufficient sequencing depth compared with the number of given molecules was attained (Fig. 1c). Then, we show experimentally that the input number of molecules and the measured output number of molecules were both consistent through a model measurement system that fulfills the two requirements. In order to fulfill these two requirements, i.e., to ensure that the digital counting system works, we introduced fixed bases within the random barcode sequence for error detection, performed barcode sequence clustering using an in-house-developed software (see Methods), and identified and removed cross-contamination between differently indexed samples and misidentifications of target sequences (templates) in the mapping process utilizing the information from our molecular barcodes. Our results show that accurate quantification of barcoded nucleic acid molecules in any given sample can be achieved with a high dynamic range (from one to more than 10^4^, potentially up to 10^15^ molecules) through appropriate barcode design (including a minimum required barcode length) and sufficient sequencing depth.

## Results and Discussion

We first investigated whether the digital counting system using random-base barcodes was able to measure the absolute number of DNA molecules in a sample accurately. We designed two types of DNA templates: six long templates (LT1–LT6) and five short templates (ST1–ST5), all of which contained random-base barcodes shown as “barcoded molecules” in Fig. 1a; the long templates have a 50-base target sequence downstream from a 50-base barcode which consists of 38 random bases and 12 fixed bases, and the short templates have an eight-base target sequence downstream from a 30-base barcode containing 24 random bases and six fixed bases (Table S1). We estimated compositions of each nucleotide at each position of the random base region in all templates based on our sequenced data which is discussed later (Fig. S1). Additionally, all templates contain common sequences at both the 5′- and the 3′- ends, which were used for PCR amplification (Tables S1 and S2). We prepared two identical samples, each containing 40,000, 40,000, 4,000, 300, 100, and 20 copies of LT1, LT2, LT3, LT4, LT5, and LT6, respectively, 20,000 copies of ST1 and ST2, and 4,000 copies of ST3, ST4, and ST5. We amplified these templates in these two samples which were distinguished by two different indices (index A and B in this study), sequenced amplicons using a MiSeq (Illumina, Inc.), and obtained 11,992,843 reads for index A and 15,373,718 for index B (Table S5). We analyzed the reads as follows (see Methods): we allowed the MiSeq to sort all reads by sample indices (A and B), mapped the sorted reads to the reference consisting of target sequences, and quantified the number of molecules for each sample index and template in a digital manner by counting the number of unique barcodes (or barcode clusters, *vide infra*) instead of counting the number of sequenced reads (i.e., amplified molecules).

**Figure 2:**
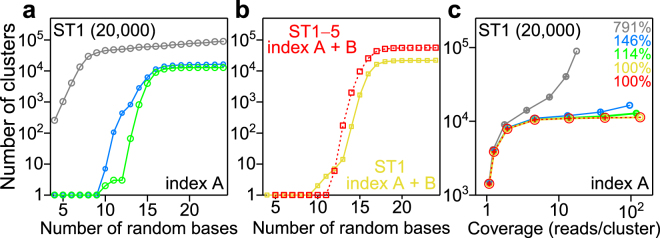
Observed innate characteristics of digital counting using random base barcodes applied to the two requirements for accurate digital quantification. (**a**) Dependency of the number of detected clusters (unique barcodes in gray) on the number of random bases. The results of ST1 are shown. Gray line, the number of unique barcodes; blue line, the number of observed clusters after clustering (*Distance* = 3); green line, the number of clusters after clustering (*Distance* = 3) and fixed base match filtering (*Fixed base number* = 6). (**b**) Dependency of the number of barcode clusters on the number of random bases. Yellow line, the number of clusters for ST1 with index A and index B after clustering (*Distance* = 3), fixed base match filtering (*Fixed base number* = 6); red dotted line, the number of barcode clusters for all short templates with index A and index B after clustering (*Distance* = 3), fixed base match filtering (*Fixed base number* = 6). (**c**) Dependency of the number of detected clusters on the sequencing coverage. The gray, blue, and green are the same as in (a). Yellow line, the number of clusters for ST1 with index A after clustering (*Distance* = 3), fixed base match filtering (*Fixed base number* = 6), and removing contaminated indices; red dotted line, the number of barcode clusters for ST1 with index A after clustering (*Distance* = 3), fixed base match filtering (*Fixed base number* = 6) and removing contaminated indices and misidentification (see Methods). Colored percentages indicate the number of clusters relative to the red at the highest coverage. Error bars represent standard deviation (n = 8).

We investigated the two requirements (above) for accurate digital quantification in the presence of errors (Figs 2 and S2): for accurate digital counting, how many random bases in the barcode are required for counting the given number of molecules in a sample, and how many reads per molecule (defined as “coverage”) are required? For the first requirement, we computationally modified the number of random bases in each template (from 4 to 38 bases for LTs, and 4 to 24 bases for STs) and determined the number of unique barcodes for each sorted index and template (Figs 2a and S2a, gray lines). The number of determined unique barcodes increased dramatically as we increased the number of random bases in the barcode, suggesting that a certain minimum number of random bases is required for quantifying a given number of molecules. We expect a plateau at 20,000 for ST1 in Fig. 2a, because even if the possible number of barcode sequences is artificially increased (by increasing barcode length), the measured number of original target sequences should not increase above the original copy number of 20,000. However, the expected plateau in the range of larger numbers of random bases was not seen and the determined number of unique barcodes increased monotonically as the number of random bases increased. For the second requirement, we again computationally modified the sequencing coverage by randomly removing a fraction of reads, and determined the number of unique barcodes using the remaining reads for each index and template (Figs 2c and S2c, gray lines). If the digital counting scheme was successful, a plateau would be observed in these plots because the number of identified unique barcodes should not depend on the coverage (i.e., sequencing depth) once the coverage reaches a sufficient level; again, we expect a plateau at 20,000 for ST1 in Fig. 2c because even upon increasing sequencing depth (i.e., the number of times each barcode is read), the measured number of original target sequences should not increase above the original copy number of 20,000. But, the expected plateau was not observed; the determined number of unique barcode increased monotonically as the coverage increased, indicating that the digital counting system in this condition was not working properly.

**Figure 3:**
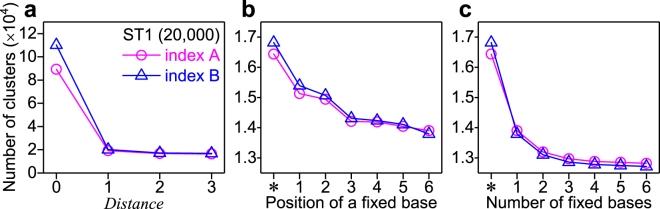
Analyses using *Distance* and fixed bases. The results of ST1 with index A (magenta circle) and index B (blue triangle) are shown. The number of random bases was 24. (**a**) Effect of clustering with different *Distance* on the number of clusters. (**b**) Dependence of the position of a fixed base with *Distance* = 3. An asterisk indicates no filtering. Only a single fixed base was used for filtering. (**c**) Dependence of the number of fixed bases with *Distance* = 3. The furthest base(s) from the sequencing primer site was used for filtering.

The reason that the plateaus were not observed in the types of figures (Figs 2a,c, S2a,c) may be explained by base changes, e.g., substitution errors and insertion-deletion (indel) errors, in the final sequenced barcode output compared to the actual barcode sequence input. In order to remove these substitution errors (possibly due to sequencing errors and/or polymerase amplification errors), we clustered the barcode sequence by using our in-house software, Nucleotide Sequence Clusterizer (see Methods). In the clustering procedure, we introduced a parameter called “*Distance*”; *Distance* indicates the number of bases which differ between two given barcode sequences. For example, if one barcode sequence becomes exactly the same sequence of another through two base changes at any two positions, the *Distance* between these two barcodes is two. So, after clustering with the parameter *Distance* = 2, all barcode sequences in a given cluster are within a *Distance* = 2 from at least one other barcode sequence (but not necessarily all other sequences) in the cluster; in essence, it may be said that the original analysis for counting the number of unique barcodes without clustering was performed by clustering with *Distance* = 0. We determined the number of barcode clusters with *Distance* = 0, 1, 2, or 3 (Figs 3a and S3a). We expected that the number of barcode clusters would approach a constant value as *Distance* increases if there was a large enough variety of barcodes for the given number of molecules, and, indeed, that tendency was seen. We confirmed that the reduction of the number of clusters down to about one fifth from *Distance* = 0 to 1 (Figs 3a, S3a) can be explained by our calculated substitution error rates for ST1, ST2, LT1, and LT2 (0.23–0.29%, Table S3) which were consistent with reported values for the Illumina MiSeq sequencer^23^. To observe the clustering effect on the two requirements for accurate digital quantification, we plotted the determined number of barcode clusters as a function of the number of random bases (Figs 2a and S2a blue lines) and coverage (Figs 2c and S2c blue lines) by using clustering with the longest *Distance* that we performed (*Distance* = 3). For both plots, more plateau-like curves were seen, but the determined number of barcode clusters still increased monotonically, particularly as coverage increased. As expected, the removal of substitution errors is not enough to make the digital counting system work.

Next, we tried to remove the effect of indel-type errors by removing sequenced reads that contained mismatched base(s) at the fixed base position(s) of the barcode sequences (Table S1); if the barcode sequence outputs contained a mismatched base at any of these fixed base positions, we knew that a base at another position in the barcode sequence had been inserted or deleted, causing the remaining bases to slide out of the designated “reading frame” as defined by the position of the fixed bases. To investigate the effect of this process on the digital counting system, we examined the position-dependence of the fixed bases in the barcode sequence; we determined the number of barcode clusters when a single fixed base is used for the removal procedure (Figs 3b and S3b). The number of barcode clusters decreased as the position of the fixed base became further away from the sequencing primer site, which is reasonable since the mismatch of a fixed base may detect indel type sequence changes that occur between the sequencing start site and the fixed base position. We also analyzed the dependence of the number of fixed bases on the determined number of barcode clusters: the fixed base(s) at the furthest position from the sequencing primer site was used (Figs 3c and S3c). The determined number of barcode clusters decreased significantly when the number of fixed bases used was small, and it became almost constant as the used number of fixed bases increased. We calculated the fixed-base mismatch rates based on our sequenced data (Table S4). To observe the mismatch removal effect on the two requirements for accurate digital quantification above, we plotted the determined number of barcode clusters as a function of the number of random bases (Figs 2a and S2a green lines) and coverage (Figs 2c and S2c green lines); we performed a mismatch removal process with the highest number of fixed bases we used (six bases for the short templates, and twelve bases for the long templates). For both type of plots (Figs 2a,c and S2a,c green lines), a plateau-like curve was apparent, indicating that indel type error removal using fixed bases resulted in more accurate digital quantification. However, the number of barcode clusters still slightly increased in the case of the coverage-dependent curve (Figs 2c and S2c green lines), which suggests that there may be additional as yet unsolved problem(s) for absolute and digital quantification.

As another problem, we also observed that cross-contamination between samples occurred, which caused the slight increase in the number of observed clusters in the plateau-like phase in Figs 2c and S2c, green lines. We simultaneously sequenced two samples that were amplified by PCR in two separate tubes, with each being labeled by a different index (A and B) through amplification primers during PCR. When we clustered barcodes with both index A and index B, we found a small fraction of barcode clusters containing both indices, which was also noticed by Jaitin and coworkers^5^. This may have occurred without cross-contamination since the barcoded templates for PCR amplification were randomly selected from the original template pool. However, since there are theoretically 2.8 × 10^14^ (=4^24^) different barcode sequences even in case of the short templates, the probability that original templates having the exact same barcode are added into two tubes for amplification is incredibly small. Therefore, either some PCR primers containing a specific index were likely to have been contaminated into the tube, the index sequences had errors, and/or index switching might have happened in the sequencing step^24^. To remove this effect, first, we mixed all reads that were sorted to two indices for each template, and performed clustering for these mixed reads. Then, when we found multiple (i.e., two) indices within one barcode cluster, we counted the barcode cluster to have the index that contained the highest number of sequenced reads (see Methods). With this process, we eventually observed a plateau in the determined number of clusters as a function of coverage (Figs 2c and S2c yellow lines). To check the first requirement for accurate digital quantification, we plotted the determined number of clusters for the sum of index A and B because the same barcode sequence was not supposed to be used for both indices (Figs 2b and S2b yellow lines). Since there was still a plateau, the used number of random bases was within the acceptable range to perform accurate digital quantification. This new functional advantage of the molecular barcode may solve the problem of “index switching”^24,25^.

Since we found cross-contamination between samples, we then looked for misidentification in the mapping process of reads to our reference. We followed a similar process as performed for the indexing issue. This time, first, we mixed all reads that were sorted to two indices and that were mapped to any template, and then performed clustering for the mixed reads. Next, when we found multiple templates and/or multiple indices within one barcode cluster, we counted the barcode cluster to the template and index which contained the highest number of sequenced reads (see Methods). With this process, we did not see significant differences in the determined number of clusters as a function of coverage (Figs 2c and S2c red dotted lines), suggesting that misidentification did not occur often in our system. To check the first requirement for accurate digital quantification, we plotted the determined number of clusters for the sum of index A, index B, and all templates because the same barcode sequence was not supposed to be used for both indices and all templates (Figs 2b and S2b red dotted lines). Since there was still a plateau, the used number of random bases was within the acceptable range to perform accurate digital counting even when we accounted for misidentification of templates. Although the misidentification effect was small in this study, this process is more important for analyses in which larger references are used, such as in RNA-Seq.^6^, because misidentification may occur more often in those cases.

To further understand what occurred during these analytical processes above, we constructed histograms of coverage for each process (Fig. S4). The histogram of counting the number of unique barcodes (without any processing above) showed a large peak containing mostly low-read clusters; these low-read clusters artificially increase the output copy number of target sequences (due to artificially created barcode sequences not present in the original sample due to sequencing errors, indel errors, etc.) as measured by our digital counting theme and must be removed in order for a system to be capable of accurate quantification (the two requirements above). After the first two processing steps, this peak dramatically decreased, suggesting that barcode sequences generated mainly by sequencing errors were removed by these processing steps.

We quantitatively investigated that the determined number of clusters (i.e., the number of molecules) depends on the process above for considering substitution errors, indel errors, cross-contamination, and misidentification in the mapping process (e.g., the number of clusters of ST1 at the highest coverage decreased from 89,329 to 11,296 for index A (Fig. 2c), and from 110,187 to 10,613 for index B (Fig. S2c)), and showed that our barcode design and computational analysis fulfilled the two requirements for accurate digital counting in the case of four specific templates: ST1, ST2, LT1, and LT2 (Fig. 2a–c and S2a–c). Next, we optimized parameters and applied these analyses for all templates that contained a wide range of copy numbers, from 20 to 40,000. Since the number of clusters determined with the parameter *Distance* = 2 was already close to a constant value (Figs 3a and S3a), we subsequently showed analyzed results with *Distance* = 2 in the main text. For the number of fixed bases, we used four fixed bases (the 16^th^, 21^st^, 24^th^ and 28^th^ fixed bases from the left in the barcode (Table S1) were used for all templates) since the determined number of clusters became close to a constant value at four fixed bases (Figs 3c and S3c).

**Figure 4:**
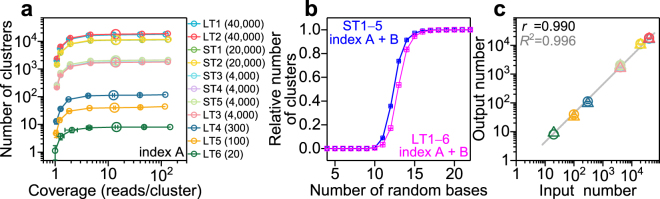
Absolute counting of each template. (**a**) Determined number of clusters as the function of coverage for index A. *Distance* = 2, *Fixed base number* = 4., *Random base number* = 20, and the effect of contaminated indices and misidentification were removed. Initial copy number of each template sequence in parentheses. (**b**) Dependency of the relative number of detected clusters on the number of random bases. *Distance* = 2, *Fixed base number* = 4, 10% of total reads sampled randomly (coverage is 13.4–20.3 for short templates, and 12.6–20.9 for long templates). (**c**) Correlation of input (x-axis), the number of molecules before PCR amplification, and output (y-axis), the result of digital counting. The output number was determined from Figs 4a and S5 at a coverage of 12.6–20.9 shown by the large symbols. Gray line indicates a fitting line with slope 1 (fixed) in log scale. Circles and triangles correspond to index A and index B, respectively. Pearson’s product-moment correlation coefficient *r* and coefficient of determination *R* ^2^ of the linear fitting are shown. Error bars represent standard deviation (n = 8).

We also considered cross-contamination of indices and misidentification of templates. By utilizing all of our quantitative analyses and insights, we were more certain that we could accurately quantify target molecules using our digital counting scheme. Based on these conditions, we again investigated the two requirements for all templates to determine the dynamic range of our digital counting system (Figs 4a,b, and S5). For coverage dependence, we used twenty random bases for clustering (Figs 4a and S5), and for the dependence on the number of random bases, we used 10% of the original total number of reads (Fig. 4b), since both parameters should still work based on the initial analyses for the four original templates (Figs 2a,c and S2a,c). When we used less than 100% of the reads for analysis, we randomly selected the reads, and showed the average and standard deviation by repeating the process eight times (Figs 4a–c and S5; see Methods). There were plateaus for all templates in both type of plots, as a function of the number of random bases and coverage, respectively, suggesting that the selected parameters allow for accurate digital quantification for templates with a high range of copy numbers.

We finally concluded that the determined number of barcodes in Figs 4a and S5 at a coverage of 12.6–20.9 (when 10% reads were sampled) corresponded to the number of templates in the sample tube before PCR amplification. Using these values, we compared the number of input (determined by optical density, see Methods) and output (determined by our digital counting method) molecules (Fig. 4c). These two values were highly correlated (Pearson’s product-moment correlation coefficient r = 0.990). The ratio of output/input was in the range of 0.32–0.45 for LTs and 0.41–0.57 for STs, which may be explained by experimental errors (e.g., systematic error in the (at most) seven-steps of template dilution in preparation for PCR amplification). This suggests that our digital counting scheme based on the parameters presented here is able to quantify the absolute copy number of nucleic acid molecules before PCR amplification. We also present the analyzed results for all templates with *Distance* = 1 and 3 (Fig. S6).

**Figure 5:**
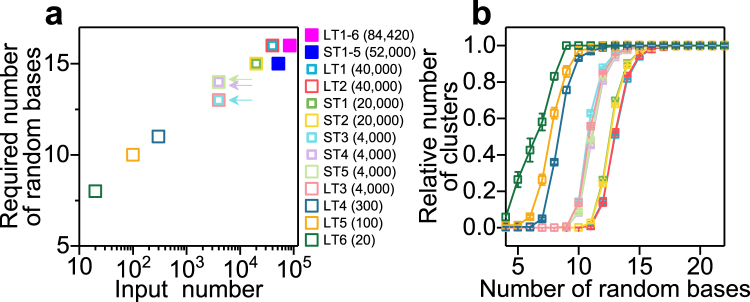
Required number of random bases for digital counting. (**a**) The x-axis indicates the input number of molecules to be measured, and the y-axis represents the number of random bases at which each curve in Figs 4b and 5b reached a relative number of clusters of 0.95. Colors correspond to the samples represented by the plots in Figs 4b and 5b. (**b**) Dependency of the relative number of barcode clusters on the number of random bases. All templates with index A and index B after clustering (*Distance* = 2), fixed base match filtering (*Fixed base number* = 4), 10% of total reads sampled randomly (e.g., coverage is 12.6–20.9). Colors correspond to the samples represented by the plots in Fig. 4a. Error bars represent standard deviation (n = 8).

Based on these results, we can now propose the required number of random bases necessary for counting the absolute number of molecules in the presence of errors (Fig. 5a): the x-axis indicates the input number of molecules to be measured, and the y-axis represents the number of random bases at which each curve in Figs 4b and 5b reached a relative number of clusters of 0.95. Figure 5b shows the dependence of the relative number of clusters on the number of random bases like in Fig. 4b, but for each template without the misidentification removal process (which did not have a significant effect on the number of clusters). We include these data in Fig. 5a in order to show more data in the lower range of the given number of molecules, and we see that, for example, sixteen random bases are required to quantify about 10^5^ molecules with greater than 95% accuracy. These values for the required number of random bases in the random-base region of the barcode were based on the randomness of nucleotide composition (17–29% for high-copy templates) which we estimated using our sequenced data as described above (Fig. S1). Actually, Fig. 5a (see arrows) suggests that ST3, which has more equal composition of four nucleotide bases, requires less random bases for accurate digital counting than ST4 and ST5, which has less equal composition (Fig. S1a). If one supposes that cross-contamination or misidentification in the mapping process is small enough and/or negligible, the required number of random bases may be lower since the same (or similar) barcode sequences can be used in different indices or genes. In the case that one must use a limited number of bases for the barcodes, we show the required number of random bases when *Distance* = 1 or 3 was used (Fig. S7). As expected, clustering with *Distance* = 1 requires less random bases, and *Distance* = 3 needs more. Basically, a larger *Distance* should be more robust to errors.

Experimentally, we showed that at most 84,420 molecules (all LTs as input) were quantified accurately using 20 random bases (Fig. 4b). This number is enough for, for instance, counting the number of RNA molecules for individual genes in transcriptome analysis. Actually, the measurable number of molecules was restricted by the capacity of the MiSeq sequencer. Since we used a maximum of 38 random bases, the required number of random bases depending on the given number of molecules (Fig. S8) suggests that about 10^15^ molecules (*Distance* = 2) may be quantified by our measurement system based on simple linear fitting for our experimentally measured data set. This dynamic range greatly exceeds the current capacity of commercially-available deep sequencers. We thereby show that the bottleneck for high dynamic quantification analysis is not limited by barcode design any more, but rather by sequencing throughput.

In summary, we have shown that digital counting using specifically designed “hybrid” molecular barcodes containing both random and fixed bases can successfully quantify the number of molecules in a given sample when coupled with appropriate barcode design, sufficient sequencing depth, and analytical methods with appropriate parameters; this allows researchers to measure the number of nucleic acid molecules with wide and high dynamic range and at low cost. Based on these results, we can now suggest the required number of random bases and fixed bases necessary for counting the given number of barcoded molecules in the presence of errors (Figs 5a and S7). We also showed quantitatively further functional advantages of the molecular barcodes: our molecular barcode was utilized in identification of sample cross-contamination (caused by physical contamination of primers, errors in indices, and/or index switching in the sequencing process) and misidentification of the target sequence in the alignment process. Actually, as described above, the former may solve a reported crucial problem in next generation sequencer platforms^24,25^. Though error effects may depend on library preparation and/or sequencing platforms, the efficacy of random-base barcodes was shown in one general application, and the strategy here for validation of the barcodes’ usage is applicable for different platforms. Moreover, since we showed the efficacy of random-base barcodes for barcoded molecules, one can evaluate the effect or efficiency of barcode attachments which may be different among applications. This study may be broadly used for digital counting methods of nucleic acid quantification using the molecular barcode, not only for counting molecules in gene expression analysis, iCLIP^8^, antibody repertoire analysis^9^, bacterial 16 S rRNA gene analysis^10,11^, and ChIP-nexus^12^, but also for cells^9,26,27^, viruses^1^, or other applications using barcodes (recently, some of these applications may be performed using commercially available instruments such as the Single Cell Sequencing Solution (Illumina, Inc., CA, USA and Bio-Rad Laboratories, Inc., CA, USA) and Chromium Single Cell 3′ Solution (10x Genomics, Inc. CA, USA)). We hope that our study accelerates systems biology based on experimentally-obtained large amounts of quantitative data.

## Methods

### Library preparation

Single-stranded DNA templates containing random bases were purchased from Integrated DNA Technologies, Inc., Coralville, IA, USA (Table S1). The concentration of each template was measured by absorbance at 260 nm using a spectrophotometer (NanoDrop 1000; Thermo Fisher Scientific Inc., MA, USA) with the absorption coefficient written in the specification sheet as provided (Integrated DNA Technologies, Inc.). Template DNAs were stocked at 50 μM in 0.1% (v/v) TWEEN 20 (Sigma-Aldrich, St. Louis, MO, USA) solution at −30 °C. To adjust the concentrations of DNA templates for amplification, all templates were diluted with water (Distilled water, deionized, sterile, NIPPON GENE CO., LTD., Toyama, Japan) and 0.1% TWEEN 20 and mixed into a PCR tube to the final copy numbers as described in the main text. Amplification was performed by PCR using MightyAmp (TAKARA BIO INC., Shiga, Japan) in a 25 μl sample with 0.3 μM of each primer (Table S2). Two tubes were prepared independently from 50 μM stock templates and distinguished by indices designed in one of the primers (Table S2). Thermal cycling (ProFlex PCR system; Themo Fisher Scientific Inc.) was performed as follows: 1 cycle of 98 °C for 2 min; 4 cycles of 98 °C for 10 sec, 60 °C for 10 sec, and 68 °C for 1 min; 19 cycles of 98 °C for 10 sec, 60 °C for 2 sec, and 68 °C for 1 min; and 1 cycle of 68 °C for 5 min; followed by a 4 °C incubation. Subsequently, amplicons were column-purified (DNA Clean & Concentrator^TM^-5; Zymo Research Corp, CA, USA) twice and their length distribution was checked with a 2100 Bioanalyzer (Agilent Technologies, Inc., CA, USA). The concentration was determined by qPCR kit (KK4602; KAPA Biosystems, Inc., MA, USA) using real-time PCR system (7500; Themo Fisher Scientific Inc.).

### Sequencing

Two indexed samples (CGCTCATT: index A, GAGATTCC: index B) were sequenced in a single run with a MiSeq sequencer (Illumina, Inc.) using the 150 cycle kit v3 (Read1: 100 cycles, Read2: 50 cycles, Index1: 8 cycles). Note that Read2 was not used for any analyses since sequences in Read2 is a part of that in Read1. Raw sequencing data used for analysis has been deposited in the GEO database GSE94895.

### Analysis

Read1 sequences were sorted by index A and B, and the fastq file for each index was generated by the MiSeq. Here, in some cases, we randomly sampled 100, 32, 10, 3.2, 1, 0.32, and 0.1 percent of the reads. The fastq files from the MiSeq were filtered by sequence length (≥34 base and ≤39 base length for short templates, and ≥ 90 base length for long templates). Read alignment for target sequences was individually performed for long and short templates using Bowtie2 v.2.2.9^28^ with target sequences of the eleven templates (Table S1 blue) as a reference. Basically, uniquely mapped reads were used for subsequent analyses. Barcode regions which were 50 base in long templates or 30 base in short templates from the 5′ end (Table S1 red) were extracted from the mapped reads. Fixed bases (maximum of six bases for short templates and twelve bases for long templates, Table S1 red) in the barcode region were used for filtering: barcodes which had at least one mismatched fixed base were removed. Then, an in-house software, Nucleotide Sequence Clusterizer, was used for clustering barcodes with *Distance* = 0, 1, 2, or 3. The number of clusters was considered to be the number of molecules before amplification. When index cross-contamination was considered, reads with index A and B were merged before clustering. In the latter, multi-mapped reads were also used for subsequent analyses. Then, after clustering, if there were clusters which contained multiple indices, the minority of reads were removed. When the number of index A reads and index B reads were identical, a coefficient of 0.5 was given for the both index A and B. Similarly, when misalignment was also considered, all template-mapped reads with both in index A and B were merged before clustering. When a read was mapped to multiple templates, a coefficient of 1/(the number of different templates) was given for each template. After clustering, if there were clusters which contained multiple target-mapped reads and/or indices, the minority of reads were removed. When the number of different template-mapped reads and/or indices were the same, a coefficient of 1/(the number of different templates and/or indices) was given for each multiple-mapped target and/or index. The number of reads in each process is shown in Table S5.

### Nucleotide Sequence Clusterizer

For clustering, an in-house software named “Nucleotide Sequence Clusterizer” was coded in C. This tool performs clustering of DNA sequences, using specified nucleotide positions of each sequence. This tool implements bounded single-linkage clustering: Initially each sequence is in a cluster of its own. When any two sequences differ from each other by not more than *D* mismatches, their clusters are merged together. Here *D* is the configurable “*Distance*” parameter. This process continues until there are no more clusters to merge, at which point the Nucleotide Sequence Clusterizer reports the number of clusters, as well as sequences in each cluster. A read that has “N” in its barcode was removed at the beginning. Nucleotide Sequence Clusterizer is made available upon request.

## Electronic supplementary material

Supplementary figures and tables
