# Supplementary material for: The efficacy and further functional advantages of random-base molecular barcodes for absolute and digital quantification of nucleic acid molecules
Source: Sci Rep. 2017 Oct 19;7:13576. doi: 10.1038/s41598-017-13529-3 (PMC5648891; doi:10.1038/s41598-017-13529-3)
Supplement: Supplementary file 1 — Supplementary figures and tables [file 41598_2017_13529_MOESM1_ESM.pdf]

Supplementary Information:

The efficacy and further functional advantages of random-base molecular barcodes for absolute and digital quantification of nucleic acid molecules

Taisaku Ogawa[1,+], Kirill Kryukov[2,+], Tadashi Imanishi[2] & Katsuyuki Shiroguchi[1,3,4,*]

[1] Laboratory for Integrative Omics, RIKEN Quantitative Biology Center (QBiC), 6-2-3 Furuedai Suita Osaka 565-0874 Japan

[2] Biomedical Informatics Laboratory, Department of Molecular Life Science, Tokai University School of Medicine,143 Shimokasuya Isehara Kanagawa 259-1193 Japan

[3] Laboratory for Immunogenetics, RIKEN Center for Integrative Medical Sciences (IMS), 1-7-22 Suehiro-cho Tsurumi-ku Yokohama 230-0045 Japan

[4] JST PRESTO, 4-1-8 Honcho Kawaguchi Saitama 332-0012 Japan

* corresponding author: katsuyuki.shiroguchi@riken.jp

[+] these authors contributed equally to this work

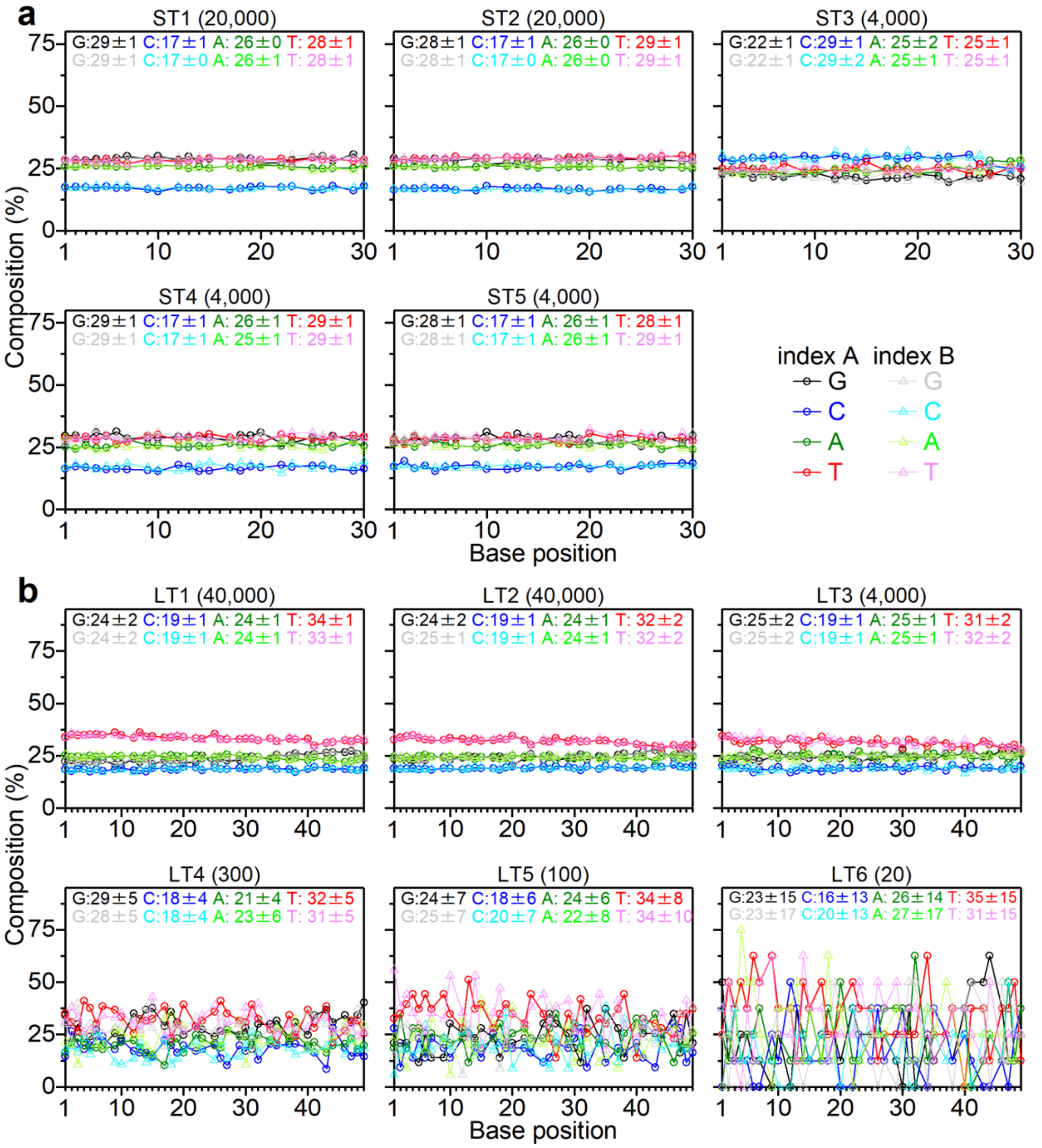

Supplemental Figure S1. The nucleotide composition at the random-base sites for STs (**a**) and LTs (**b**). The composition was calculated as relative frequency of each nucleotide at random-base positions using the highest-number barcode sequences in each cluster which was used in Figs. 2c and S2c red. The numbers in parentheses are the number of input molecules. Base position in x-axis represents the nucleotide position from 5'-end shown in Table S1. Averaged compositions for all positions are shown in each graph (mean ± standard deviation (n = 24 for **a**, 38 for **b**)).

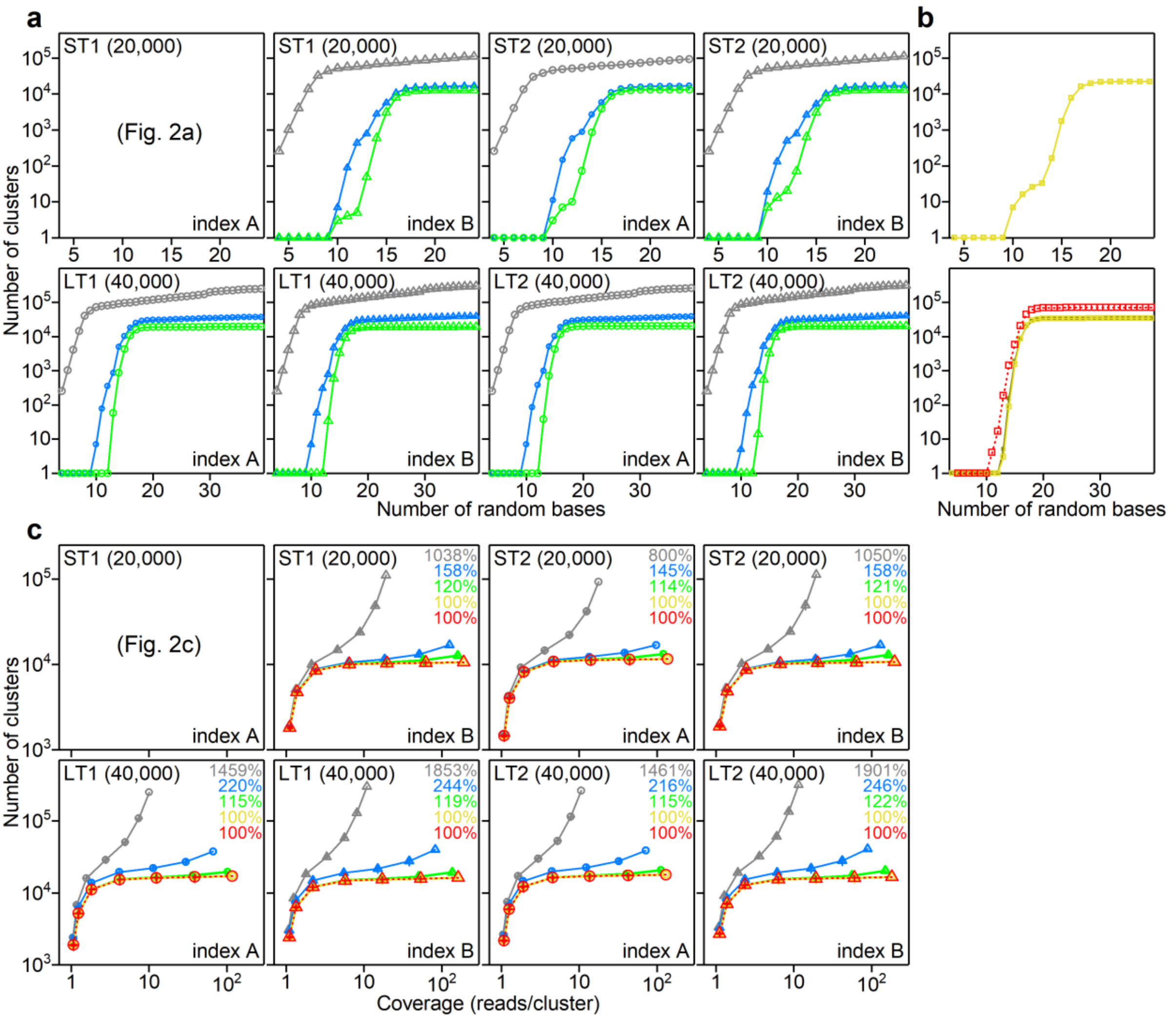

Supplemental Figure S2. Observed innate characteristics of digital counting. (**a**) Dependency of the number of detected clusters (unique barcodes for gray) on the number of random bases for ST1, ST2, LT1, and LT2. The plot colors are the same as in Figs 2a and 2b. (**b**) Dependency of the number of barcode clusters on the number of random bases. Yellow lines, the number of clusters for ST2 (upper panel) and LT2 (lower panel) with index A and index B after clustering (*Distance* = *3*), fixed base match filtering (*Fixed base number* = *6* (for ST2), and *12* (for LT2)), and removing contaminated indices; dark yellow lines, the number of clusters for LT1 (lower panel) with index A and index B after clustering (*Distance* = *3*), fixed base match filtering (*Fixed base number* = 12), and removing contaminated indices; red dotted lines, the number of barcode clusters for all long templates with index A and index B after clustering (*Distance* = *3*), fixed base match filtering (*Fixed base number* = 6) and removing contaminated indices and misidentification. (**c**) Dependency of the number of detected clusters on the sequencing coverage for ST1, ST2, LT1, and LT2. The plot colors are the same as in **a** and **b** except for dark yellow. Colored percentages indicate each number of clusters relative to red at its highest coverage. Error bars represent standard deviation (n = 8).

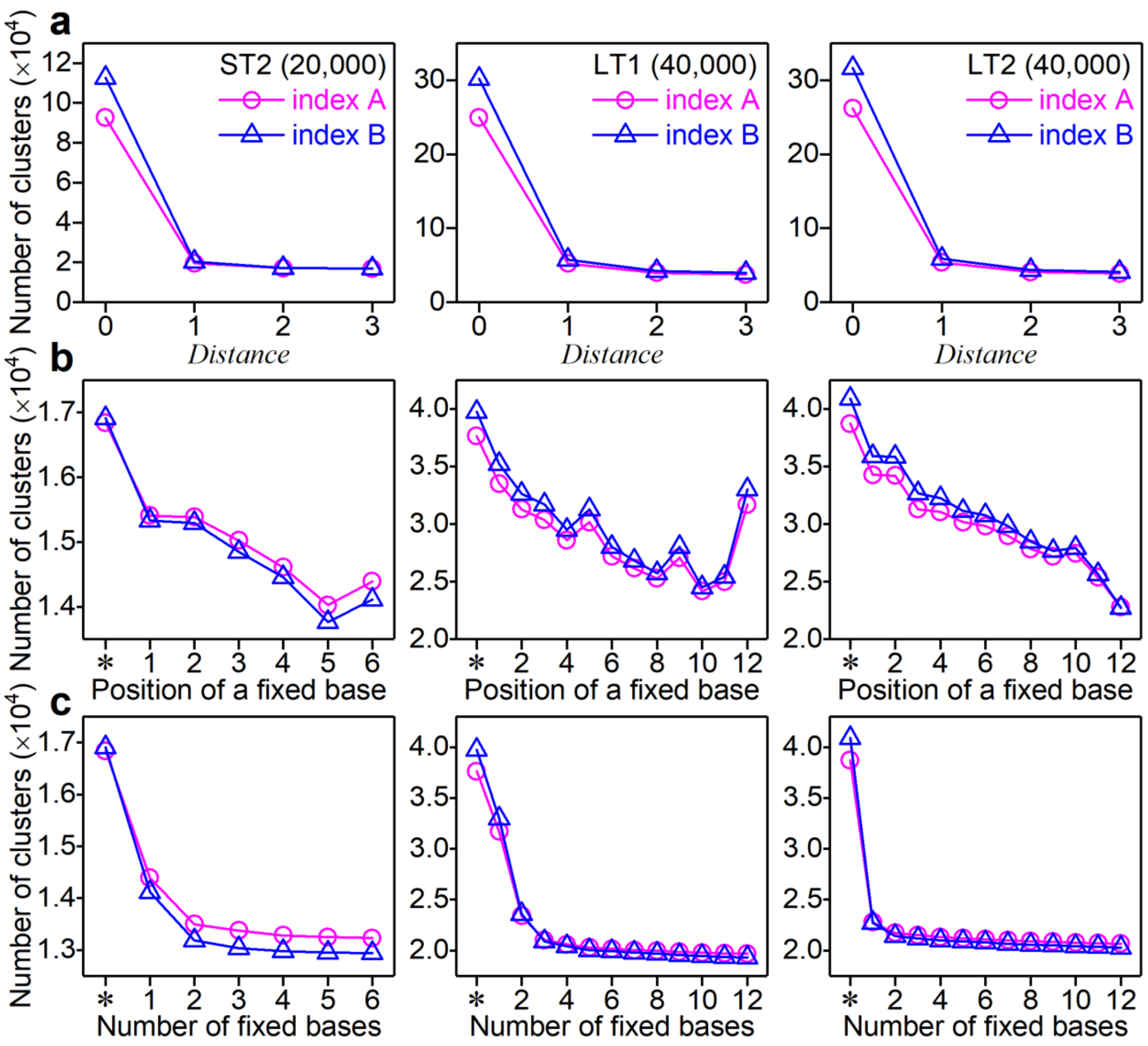

Supplemental Figure S3. Analyses using *Distance* and fixed bases for ST2, LT1, and LT2. (**a**) Effect of clustering with different *Distance* parameters on the number of clusters, the same as Fig. 3a but for ST2, LT1, and LT2. (**b**) Dependence of the position of a fixed base, the same as Fig. 3b but for ST2, LT1, and LT2. An asterisk indicates no filtering. (**c**) Dependence of the number of fixed bases, the same as Fig. 3c but for ST2, LT1, and LT2.

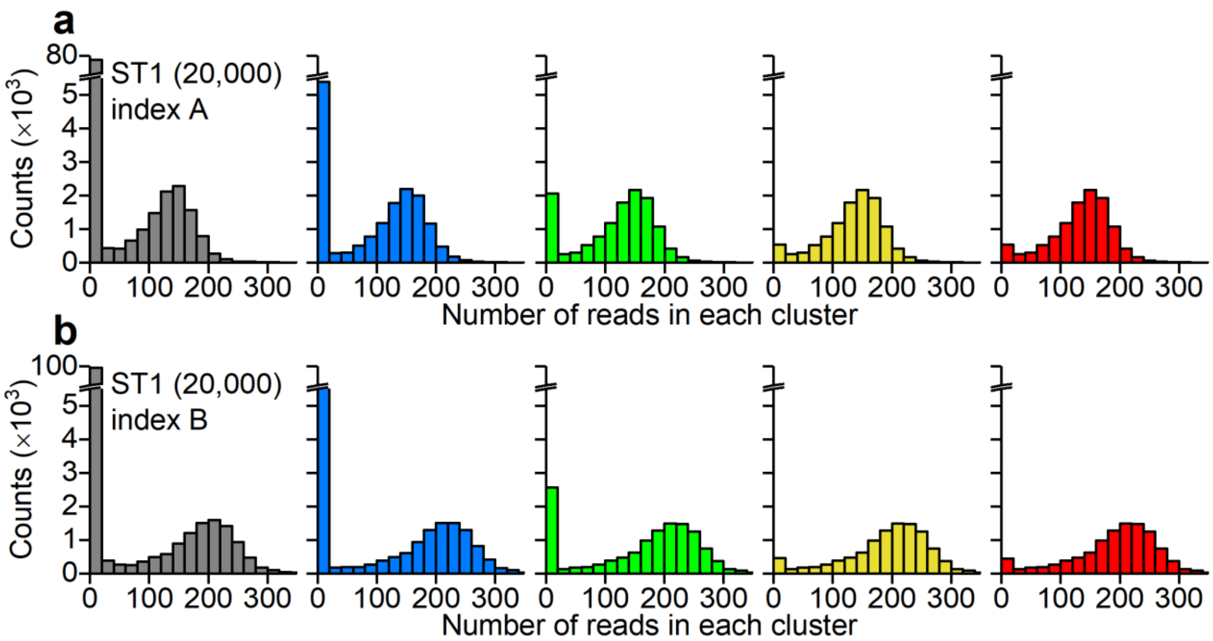

Supplemental Figure S4. Histogram of the number of reads in each cluster for ST1 with index A (**a**) and index B (**b**). Colors correspond to the samples represented by the plots in Fig. 2c.

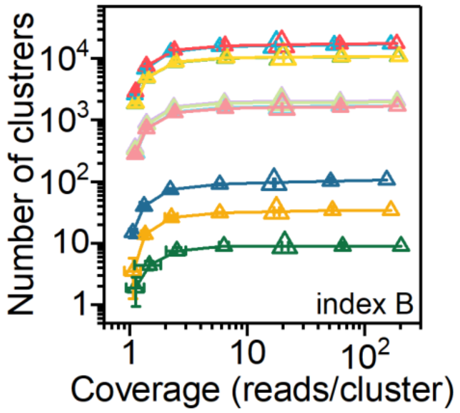

Supplemental Figure S5. Absolute counting of each template. Determined number of clusters as the function of coverage, the same as Fig. 4a, but for index B (triangles). Error bars represent standard deviation (n = 8).

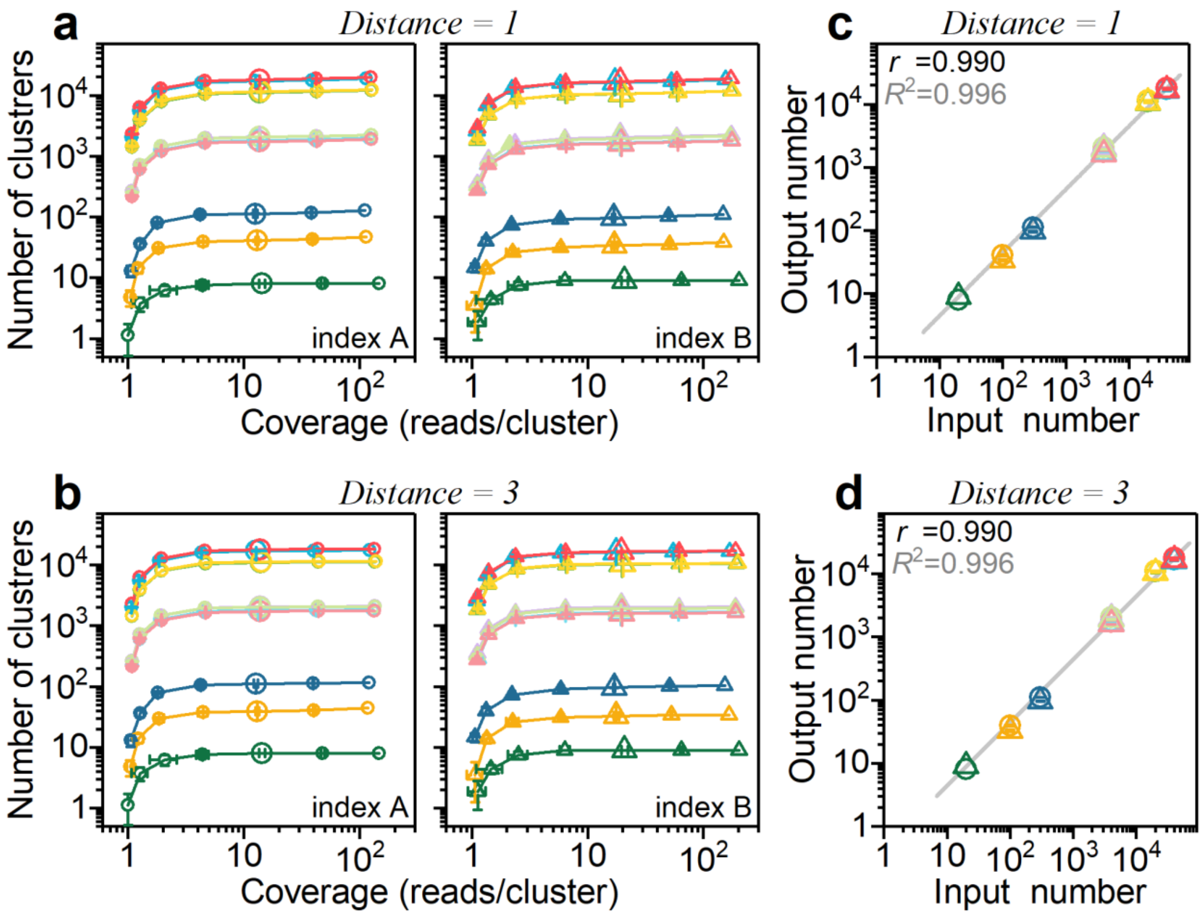

Supplemental Figure S6. Absolute counting of each template. (**a**, **b**) Determined number of clusters as the function of coverage, the same as Figs. 4a and S5, but with different *Distance* (*1* for **a**, *3* for **b**). (**c**, **d**) Correlation of input and output, the same as Fig. 4c, but with different *Distance* (*1* for **c**, *3* for **d**). Error bars represent standard deviation (n = 8).

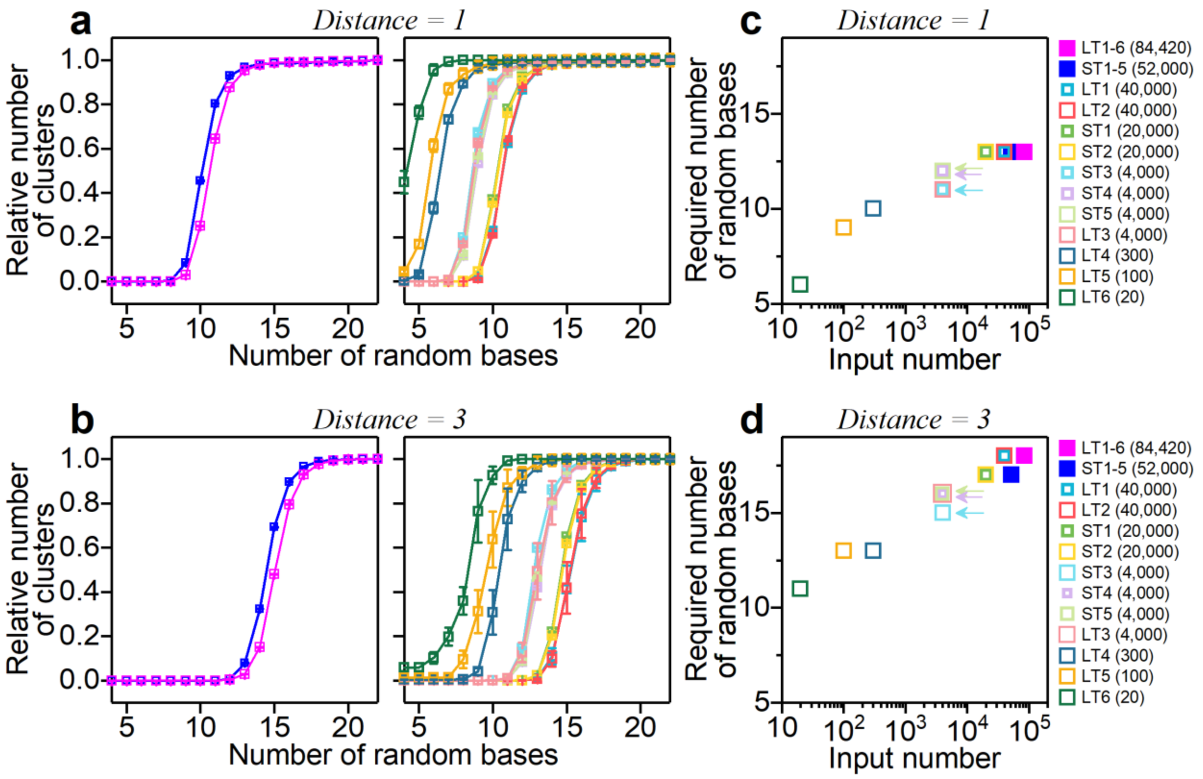

Supplemental Figure S7. (**a**, **b**) Dependency of the relative number of barcode clusters on the number of random bases, the same as Figs. 4b and 5b, but with different *Distance* (*1* for **a**, *3* for **b**). Error bars represent standard deviation (n = 8). (**c**, **d**) Required number of random bases for digital counting, the same as Fig. 5a, but with different *Distance* (*1* for **c**, *3* for **d**).

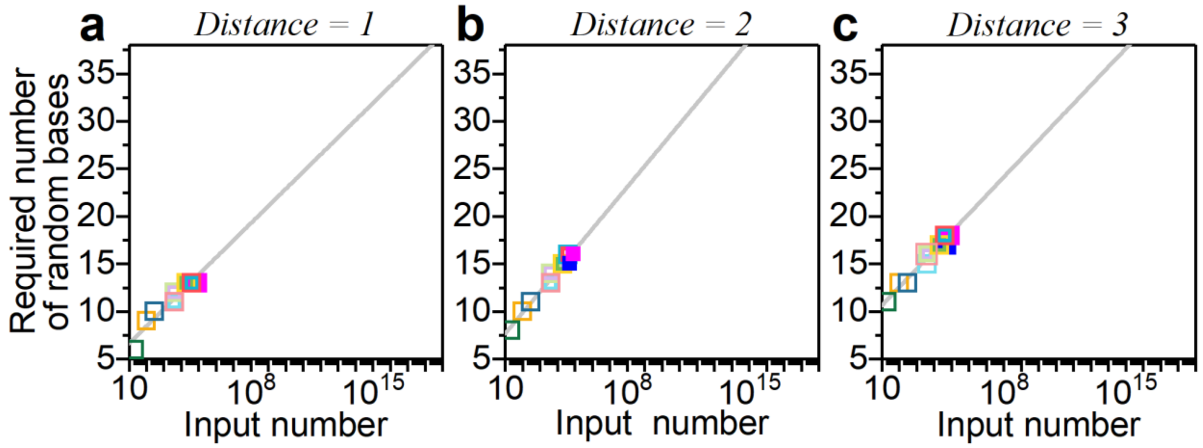

Supplemental Figure S8. Estimated required number of random bases for digital counting. The same plot as Figs. 5a (**b**), S7c (**a**) and S7d (**c**) except for a linear fit curve in log scale (R^2 = 0.912 (**a**), 0.966 (**b**), 0.957 (**c**)) up to 38 random bases (the longest in this study).

Supplemental Table S1. Barcode design and input number of molecules

Template name		Sequence[a]	Input number	Random bases mixing option[b]	Type of DNA Oligo[c]
ST1-5		ACTCTTTCCCTACACGACGCTCTTCCGATCT-barcode-target-AGATCGGAAGAGCACACGTCTGAACTCCAGT			
ST1	barcode	NNNNNNNGNNTNNNNCNNNNANNGNNNANN	20,000	"Hand-mixed" G:C:A:T=29:21:25:25	"Custom" "Standard Desalting"
	target	ATTCAGAA			
ST2	barcode	NNNNNNNCNNANNNNGNNNNTNNCNNNTNN	20,000	"Hand-mixed" G:C:A:T=29:21:25:25	"Custom" "Standard Desalting"
	target	GAATTCGT			
ST3	barcode	NNNNNNNANNCNNNNTNNNNGNNANNNCNN	4,000	"Hand-mixed" G:C:A:T=25:25:25:25	"Custom" "Standard Desalting"
	target	ATTACTCG			
ST4	barcode	NNNNNNNANNCNNNNTNNNNGNNANNNCNN	4,000	"Hand-mixed" G:C:A:T=29:21:25:25	"Custom" "Standard Desalting"
	target	CGCTCATT			
ST5	barcode	NNNNNNNTNNGNNNNANNNNCNNTNNNGNN	4,000	"Hand-mixed" G:C:A:T=29:21:25:25	"Custom" "Standard Desalting"
	target	GAGATTCC			
LT1-6		ACACTCTTTCCCTACACGACGCTCTTCCGATCT-barcode-target-AGATCGGAAGAGCACACGTCTGAACTCCAGTCAC			
LT1	barcode	NNNNNNNTNNGNNNNANNNNCNNTNNNGNNNNGNNCNNTNNCNNGNNNNA	40,000	"Machine-mixed"	"Ultramer" "Standard Desalting"
	target	AGCCACCACACCGCCTCGCCCTGGAACCTCAGCCCCTTCTCCAAGACGTC			
LT2	barcode	NNNNNNNGNNTNNNNCNNNNANNGNNNANNNNANNANNGNNTNNCNNNNC	40,000	"Machine-mixed"	"Ultramer" "Standard Desalting"
	target	TGAGTTGTCAGGTAAAGATGAGCCTTCCAGCTACATTTGCACAACATGCA			
LT3	barcode	NNNNNNNANNCNNNNTNNNNGNNANNNCNNNNCNNTNNANNGNNANNNNT	4,000	"Machine-mixed"	"Ultramer" "Standard Desalting"
	target	GGCTCTCCGGGGCCTCTGTCCGTTTACCCTCCGGCTTCATCCTCTTCTCT			
LT4	barcode	NNNNNNNGNNTNNNNCNNNNANNGNNNANNNNANNANNGNNTNNCNNNNC	300	"Machine-mixed"	"Ultramer" "Standard Desalting"
	target	CTGTCCCCGGTCCCCAGCTCCCCAGGACTCACCCTCGTATCTTCTGTTGC			
LT5	barcode	NNNNNNNANNCNNNNTNNNNGNNANNNCNNNNCNNTNNANNGNNANNNNT	100	"Machine-mixed"	"Ultramer" "Standard Desalting"
	target	GTACTTACCCGGAGAAGGCCGCTGCCCCAGCCCCGTTCCTTCCGATGACA			
LT6	barcode	NNNNNNNCNNANNNNGNNNNTNNCNNNTNNNNTNNGNNCNNANNTNNNNG	20	"Machine-mixed"	"Ultramer" "Standard Desalting"
	target	CAGTCATGTGAACTGGACGTTAAAGGTGGATATTTACATAGTGTTGACGT			

[a] Black, PCR primer priming sites; red, random region including random bases and fixed bases; blue, target sequences. In addition, fixed bases between random bases help to avoid long homopolymer barcodes which may have lower amplification efficiency. The 5' ends of ST1-5 were anime-modified.

[b] Machine-mixed, all bases are supposed to be mixed at equal molar concentrations by a machine; hand-mixed, each base is mixed by hand at user-defined ratio, where each coupling efficiency is considered (see details at website of Integrated DNA Technologies, Inc.). The designed ratio was not equal (i.e., 25%) in ST1, 2, 4, and 5 in order to see accuracy of hand-mixed.

[c] See details at website of Integrated DNA Technologies, Inc.

Supplemental Table S2. Primer sequences for library preparation.

Primer	Sequence	Length
Fw primer	AATGATACGGCGACCACCGAGATCTACACTATAGCCT ACACTCTTTCCCTACACGACGCTCTTCCGATCT	70
Rv primer1	CAAGCAGAAGACGGCATACGAGAT**AATGAGCG**GTGACTGGAGTTCAGACGTGTGCTCTTCCGATCT	66
Rv primer2	CAAGCAGAAGACGGCATACGAGAT**GGAATCTC**GTGACTGGAGTTCAGACGTGTGCTCTTCCGATCT	66

Underlined, index sequences (A, Rv primer1; B, Rv primer2). All three primers were PAGE purified.

Supplemental Table S3. Calculated substitution error rates.

Template name	Index	Input number	$L_{barcode}$ (bases)[a]	N_{read} (reads)[b]	$N_{sub\text{-}error}$ (substitutions)[c]	E_{sub} (substitutions/base)[d]
ST1	A	20,000	30	1,579,407	111,855	0.00236
	B			2,115,083	153,233	0.00241
ST2	A	20,000	30	1,642,668	117,783	0.00239
	B			2,185,838	157,357	0.00240
LT1	A	40,000	50	2,507,420	359,984	0.00287
	B			3,277,766	472,341	0.00288
LT2	A	40,000	50	2,776,540	383,459	0.00276
	B			3,641,075	504,023	0.00277

[a] Barcode length.

[b] The number of sequenced reads that did not contain "N" in their barcodes.

[c] Sum of the number of bases which differ from the highest-read barcode sequence in each cluster generated with *Distance = 3*.

[d] Substitution error rate is calculated as:

$$E_{sub} = N_{sub\text{-}error} / (N_{read} \times L_{barcode})$$

We confirmed that the reduction of the number of clusters down to about one fifth from *Distance = 0* to *1* (Figs. 3a, S3a) was consistent with our calculated substitution error rates. For a simple explanation, we suppose that in each cluster 4 reads on average have a single substitution error at different positions of barcodes respectively (i.e., 4 of the 5 unique sequences within a single cluster of *Distance = 1* are the result of a substitution error; assuming each of these sequences was read exactly once, within a single cluster of *Distance = 1* there are 4 total substitution errors, while the entirety of the rest of the reads within this given cluster contains no substitution errors of any kind), which means that the number of cluster reduces down to one fifth from *Distance = 0* to *1*. Based on this assumption, we calculated the error rate for the short templates when coverage is 100 as an example.

Coverage for ST1: 100 (reads/cluster) (the highest coverage in Fig. 2c)

The averaged number of substitution errors in each cluster: 4 (bases/cluster)

Total length of barcodes: 30 (bases/read) × 100 (reads/cluster)

Substitution error rate = 4 / (30 × 100) = 0.0013

Actually, the coverage (100) used in this example is close to the actual number in the condition of Fig. 3a (coverage: 82.4, *Distance = 1*). Based on this model, our calculated error rates may reasonably explain the reduction of the number of clusters from *Distance = 0* to *1* in Fig 3a and S3a.

Supplemental Table S4. Calculated fixed-base mismatch rates.

Template name	Index	Input number	Fixed-base mismatch rate (fixed-base mismatched read/total read)[a]
ST1	A	20,000	0.0160
	B		0.0160
ST2	A	20,000	0.0123
	B		0.0125
LT1	A	40,000	0.0411
	B		0.0424
LT2	A	40,000	0.170
	B		0.115

[a] These were calculated for 38 base length (ST1 and ST2) and 100 base length (LT1 and LT2). *Fixed base number* = 6 (ST1 and ST2) and 12 (LT1 and LT2). These errors may include both indel error and synthesis error of templates purchased from the oligonucleotide synthesis company.

Supplemental Table S5. The number of reads in each process.

**Index**	A				B			
**Template name**	ST1-5		LT1-6		ST1-5		LT1-6	
**MiSeq pass filter**	11,992,843				15,373,718			
**Separation by read length**	4,175,940	100%	7,741,172	100%	5,589,729	100%	9,691,528	100%
**Uniquely mapped**	4,051,451	97%	5,569,701	72%	5,419,415	97%	7,300,023	75%
**Fixed base match filtering**	3,912,845	94%	4,595,705	59%	5,233,818	94%	6,010,162	62%
**Removing contaminated indices**	3,906,577	94%	4,587,852	59%	5,225,911	93%	6,000,049	62%
**Removing contaminated indices and misidentification***	3,906,581	94%	4,588,431	59%	5,225,894	93%	6,000,906	62%

* This fraction may be higher than that in "Removing contaminated indices" (see Methods).
